# Heart Failure in Older Adults: Real-World Outcomes and Patient Profiles by Admission Service and Sex

**DOI:** 10.3390/jcm15114062

**Published:** 2026-05-24

**Authors:** Clara Bonanad, Claudio Rivadulla, Francisca Esteve-Claramunt, Daznia Bompart, Guillermo Barreres, Carles Muñoz-Alfonso, Daniela Maidana, Enrique Rodriguez-Borja, Gema Torres, Georgiana Zaharia, Sergio Garcia-Blas

**Affiliations:** 1 Cardiology Department, Hospital Clínico Universitario de Valencia, 46010 Valencia, Spain; 2 INCLIVA Health Research Institute, 46010 Valencia, Spain; paqui.esteve@hotmail.com (F.E.-C.); dazniabompart@gmail.com (D.B.); gbarreres@incliva.es (G.B.); cmunoz@incliva.es (C.M.-A.); danielamaidana@campus.fmed.uba.ar (D.M.); gzaharia@incliva.es (G.Z.); 3 Centro de Investigación Biomédica en Red (CIBER)-Cardiovascular, 28029 Madrid, Spain; 4 Department of Medicine, University of Valencia, 46010 Valencia, Spain; 5 Department of Nursing, University of Valencia, 46010 Valencia, Spain; 6 Laboratory Department, Hospital Clínico Universitario de Valencia, 46010 Valencia, Spain; rodriguez_enr@gva.es; 7 Documentary Department, Hospital Clínico Universitario de Valencia, 46010 Valencia, Spain; gtorresfisio@gmail.com

**Keywords:** heart failure, older patients, hospital admission, internal medicine, cardiology, sex differences

## Abstract

**Objectives**: This study aims to describe real-world clinical profiles, treatment patterns and one-year outcomes of older adults hospitalized for acute heart failure (HF) across different admission services, and to examine whether sex influences these characteristics and outcomes. **Methods**: We performed a retrospective study including 1226 patients aged ≥80 years hospitalized for acute HF between 2018 and 2021. Patients were categorized by admitting service (cardiology [CAR] or internal medicine [IM]) and sex. The primary endpoint was all-cause mortality at 12 months, with secondary endpoints including HF readmission and a composite of death or HF readmission. **Results**: The mean age was 87.4 ± 4.4 years, 65.8% were women, and 80% presented HF with preserved ejection fraction (HFpEF). Admission services reflected differing patient profiles: individuals admitted under IM were older, predominantly female, and more frequently presented with HFpEF and worse functional class, while CAR admissions included a higher proportion of patients with ischemic disease. Use of guideline-directed medical therapy varied according to clinical characteristics and admitting service. At 12 months, mortality and the composite endpoint differed across admission services, whereas HF readmission rates were similar. Sex-stratified analyses showed no difference in all-cause mortality or in the composite endpoint, but women experienced more frequent HF readmissions. **Conclusions**: Among very old adults hospitalized for acute HF, clinical profiles, therapeutic patterns, and outcomes differ according to patient characteristics and hospital admission pathways. Sex also shapes clinical presentation and readmission patterns. These findings highlight the importance of harmonized, multidisciplinary, and sex-sensitive HF care pathways to address the diverse needs of an aging HF population.

## 1. Introduction

Despite remarkable therapeutic advances, heart failure (HF) remains a leading cause of morbidity, mortality, and healthcare expenditure worldwide, with a prevalence and impact that rise with age [1,2,3]. In Spain, HF represents the most frequent cause of hospitalization for people over 65, accounting for 3–5% of all hospital admissions and generating significant clinical and organizational challenges [4,5,6,7,8,9]. Moreover, recent European registries highlight high one-year mortality and marked regional heterogeneity after acute HF, reflecting differences in population characteristics and local care structures [10,11].

HF in older patients is a clinical challenge. Beyond cardiac dysfunction, conditions such as frailty, multimorbidity, social and functional limitations, and polypharmacy are nearly universal in this population [12,13,14,15,16]. Elderly patients, particularly those over 80 years old, are underrepresented in clinical trials, which limits the validity of research findings [10,11,12,13]. As a result, extrapolating guideline recommendations to routine practice in octogenarians is challenging, and this contributes to variability in the use of guideline-directed medical therapy (GDMT), including renin–angiotensin–aldosterone system (RAAS) inhibitors, β-blockers, mineralocorticoid receptor agonists (MRAs), and sodium–glucose cotransporter-2 (SGLT2) inhibitors [6,14,16].

Sex differences further shape the HF experience: women, who represent most octogenarian HF patients, more often present with HF with preserved ejection fraction (HFpEF), anemia, and multiple comorbidities [17,18,19]. These differences may influence treatment patterns and clinical outcomes. In many hospitals, older adults with HF are typically admitted to either cardiology (CAR) or internal medicine (IM) services [16]. These pathways often reflect the patient’s overall clinical profile, comorbidity burden, and resource availability [20]. Previous studies have described differences in treatment patterns and outcomes across admission services; however, data specifically addressing very elderly patients remain limited [20,21,22,23]. Against this background, we conducted a real-world study of patients aged ≥80 years and hospitalized for acute HF. The objective was to characterize clinical profiles, treatment patterns, and outcomes over a 12-month period according to admission service and sex. This approach aims to enhance understanding of diversity in clinical presentations and care pathways to inform integrated, patient-centered HF management for this growing population.

## 2. Materials and Methods

### 2.1. Study Design and Setting

We conducted a retrospective cohort study of consecutive patients admitted to a tertiary hospital in Valencia (Spain) between January 2018 and December 2021. Patients were identified through electronic hospital records, and eligibility was confirmed through a chart review.

### 2.2. Study Population

**Figure 1:**
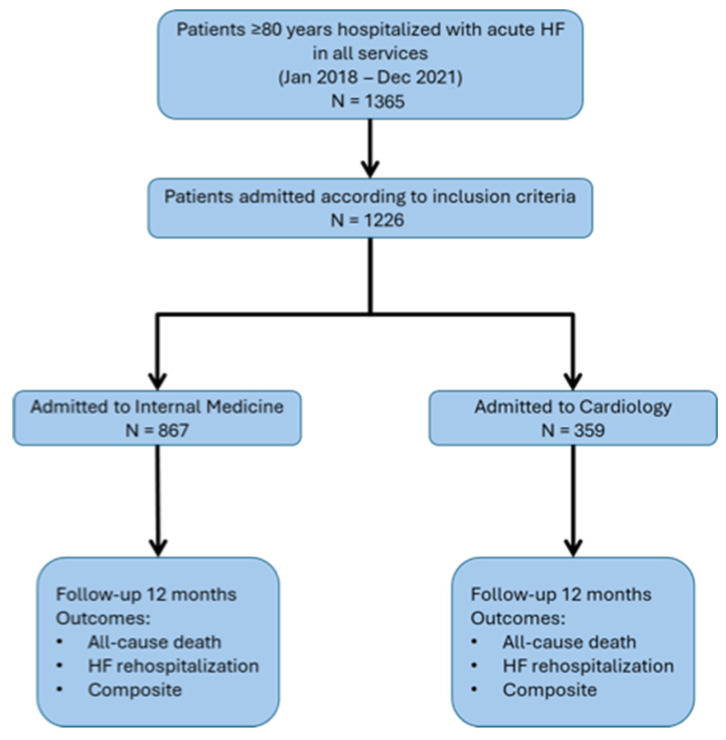
CONSORT flow diagram. Flowchart showing the selection and stratification of patients older than 80 years old and hospitalized with acute HF during January 2018 and December 2021. CONSORT: Consolidated Standards of Reporting Trials; HF: heart failure; N: number of patients.

The study included 1226 patients aged ≥80 years admitted either to the cardiology or internal medicine ward with a primary discharge diagnosis of acute HF, confirmed according to the European Society of Cardiology diagnosis criteria (presence of typical symptoms and signs of HF, together with objective evidence of cardiac dysfunction) [24]. A Consolidated Standards of Reporting Trials (CONSORT) diagram of this study is shown in Figure 1.

### 2.3. Baseline Characteristics

Demographic and clinical baseline characteristics were extracted at the time of admission. These included age and sex; functional status according to the New York Heart Association (NYHA) functional classification; left ventricular ejection fraction (LVEF), categorized as preserved (≥50%) or reduced (<50%); and renal function assessed by the Modification of Diet in Renal Disease equation, classified as impaired (<60 mL/min/1.73 m^2^) or preserved (≥60 mL/min/1.73 m^2^). Relevant comorbidities, such as hypertension, diabetes mellitus, atrial fibrillation, ischemic heart disease and valvular disease, were recorded.

### 2.4. Treatment Variables

Pharmacological treatment data were obtained from discharge prescriptions and in-hospital medication records. GDMTs were defined as the use of any of the following: angiotensin-converting enzyme inhibitors, angiotensin receptor blockers, angiotensin receptor–neprilysin inhibitors (ARNis), β-blockers, MRAs, SGLT2 inhibitors, and loop diuretics. Additional therapies of interest, including antiplatelets, oral anticoagulants, nitrates, and statins, as well as the use of an implantable cardioverter-defibrillator or cardiac resynchronization therapy, were also recorded. Treatment intensity was assessed by comparing prescription patterns between admission services.

### 2.5. Study Hypothesis and Outcomes

We hypothesized that differences in HF would vary by treatment, sex and local care structure. Follow-up was performed for 12 months after the first hospitalization through linked hospital discharge and mortality records. The primary outcome was all-cause mortality at 12 months after first hospitalization. Secondary endpoints included HF readmission and a composite endpoint of all-cause death or HF readmission within 12 months.

### 2.6. Statistical Analysis

Continuous variables were first assessed for normality by the Kolmogorov–Smirnov test. Variables with a normal distribution are presented as mean ± standard deviation (SD) and compared using Student’s *t*-test, while non-normally distributed variables are expressed as median with interquartile range (IQR) and compared using the Mann–Whitney U test.

Categorical variables are expressed as absolute counts and percentages, with between-group differences assessed using the chi-square test or Fisher’s exact test, as appropriate.

Survival analyses were performed using Kaplan–Meier curves, with a log-rank test to compare the CAR and IM groups. Multivariable Cox proportional hazards models were used to estimate hazard ratios (HRs) and 95% confidence intervals (CIs), adjusting for prespecified covariates: age, sex, renal function (Modification of Diet in Renal Disease equation), LVEF, NYHA functional class, ischemic etiology, diabetes mellitus, history of AF, previous HF admission, and systolic blood pressure. Notably, only patients without missing data in these variables were included in the model (*n* = 580). For the analyses of first readmission, death was taken as a competing event, and the Fine–Gray method was used to modify the Cox proportional hazard model.

All statistical tests were two-sided, and a *p*-value < 0.05 was considered statistically significant (* *p*-value < 0.05; ** *p*-value < 0.01; *** *p*-value < 0.001). Statistical analyses were performed using SPSS v.17.0 (Chicago, IL, USA) and R studio v.4.6.0. (R Foundation for Statistical Computing, Vienna, Austria).

### 2.7. Ethical Considerations

The study was conducted in accordance with the Declaration of Helsinki and the Good Clinical Practice guidelines issued by the Spanish regulatory authorities and was evaluated and approved by the ethics committee of the Hospital Clínico Universitario de Valencia. Given the retrospective design and use of anonymized data, informed consent was waived.

## 3. Results

### 3.1. Baseline Characteristics

A total of 1226 patients aged ≥80 years hospitalized for HF were included in the study. The mean age of the cohort was 87.4 ± 4.4 years, with women representing nearly two-thirds of all patients (65.8%), who were slightly older than men (87.8 ± 4.4 vs. 86.6 ± 4.2 years, *p* < 0.001).

Overall, most patients presented HFpEF (80%), a pattern particularly frequent among women (88.2%). Sex-stratified analysis showed that women had a slightly greater burden of hypertension and a higher percentage of diabetes, while men more often exhibited ischemic heart disease, HF with reduced ejection fraction (HFrEF), and impaired renal function. Table 1 summarizes baseline characteristics stratified by admission service and sex. Sex patterns were consistent across both admission services.

Regarding admittance services, 61% of patients were hospitalized under IM and 39% under CAR. Those admitted to IM were significantly older (88.3 ± 4.4 vs. 85.0 ± 3.5 years, *p* < 0.001), more often female (69% vs. 31%, *p* < 0.001), and more frequently presented with HFpEF (86.3% vs. 72.8%, *p* < 0.001) and worse functional status (higher NYHA > II; 57.1% vs. 39.6%, *p* < 0.001). Patients admitted under CAR had a higher prevalence of ischemic heart disease and iron deficiency (Table 1).

### 3.2. Treatment Patterns

Cardiology patients were more frequently discharged on GDMT as observed in Table 2. Across the overall cohort, 53.3% received β-blockers, 12.5% angiotensin-converting enzyme inhibitors, 23% angiotensin receptor blockers, 5.4% ARNis, 19.6% MRAs, 13.8% SGLT2 inhibitors, and 72.3% loop diuretics.

When stratified by admission service (Table 2), CAR patients were significantly more likely to receive nearly all evidence-based HF medications than IM patients, including β-blockers (71% vs. 45.9%, *p* < 0.001), MRAs (29% vs. 15.7%, *p* < 0.001), SGLT2 inhibitors (25.3% vs. 9%, *p* < 0.001), and ARNis (12.8% vs. 2.3%, *p* < 0.001). The use of loop diuretics (88% vs. 65.9%, *p* < 0.001), statins (57.9% vs. 32.3%, *p* < 0.001), and antithrombotic agents was also higher in the cardiology group. Between sexes, women were less frequently prescribed angiotensin receptor blockers, ARNIs, and statins, while men showed greater exposure to antiplatelet drugs (Table 2).

Table 3 explores sex-specific prescribing patterns within each hospital service. Admission under CAR markedly increases the likelihood of receiving evidence-based therapies in both men and women, corroborating the overall statistics from the admission service observed in Table 2. In CAR, treatment rates were substantially higher for both sexes. However, men were more likely to receive ARNis (18.7% vs. 8.6%, *p* = 0.005) and MRAs (35.3% vs. 24.4%, *p* = 0.002), while women had higher prescription rates of β-blockers (76.1% vs. 64%, *p* = 0.01). Loop diuretics were prescribed in more than 88% of patients regardless of sex. In contrast, within the IM service, women were more frequently prescribed β-blockers (48.8% vs. 39.4%, *p* = 0.01) and MRAs (17.4% vs. 11.9%, *p* = 0.04) compared with men. Men were slightly more likely to receive ARNis (3.7% vs. 1.7%, *p* = 0.06, not statistically significant). Discharge medication stratified by sex and service for patients with HFrEF is shown in Table 4; with HFmrEF in Table 5; and with HFpEF in Table 6.

When all groups were combined, differences between services remained highly significant across all major HF drug classes (*p* < 0.001), highlighting that treatment disparities are driven by both the type of service and patient sex.

### 3.3. One-Year Outcomes by Admission Service

At one-year follow-up, cumulative all-cause mortality was significantly higher among IM patients with 52% (95% CI) (48.3–55.7) vs. cardiology patients with 30% (25.1–34.9) (HR 2.15; 95% CI 1.72–2.69; *p* < 0.001). After adjustment for age, sex, renal function, LVEF, NYHA class, diabetes, blood pressure and ischemic etiology, IM admission remained independently associated with higher all-cause mortality (HR 1.91; 95% CI 1.41–2.60; *p* < 0.001; Table 7). As HF readmission and death are competitive events, the cumulative risk function was used to test whether admission service was related to these events. HF readmission alone did not differ significantly between services, despite a numerical excess in IM patients (first HF admission in IM: 9.8% (95% CI) (6.7–12.9); cardiology: 5.9% (95% CI) (3–8.8)) (Table 7). However, there was a statistically significant association with the composite outcome of death by any cause or HF readmission (HR 1.82; 95% CI 1.38–2.42; *p* < 0.001; Table 7) between admission services (IM: 57.1% (53.4–60.8); cardiology: 35.8 (31.2–40.4)).

### 3.4. Sex Differences

Sex-stratified analysis showed that the primary endpoint (all-cause mortality) yielded no statistically significant differences in the multivariate adjusted by admission service, age, renal function, LVEF, NYHA, ischemic etiology, diabetes mellitus, systolic blood pressure, previous history of atrial fibrillation and previous HF admission analysis (HR 0.91; 95% CI 0.68–1.22; *p* = 0.56; Table 7). Similar results were obtained when analyzing HF readmission (as a competitive event with death) and the composite endpoint of death or first HF readmission (Table 7).

## 4. Discussion

In this large real-world cohort of octogenarian patients hospitalized for HF, we found differences in clinical profiles, therapeutic patterns, treatment, and outcomes. Rather than reflecting performance differences between departments, these variations appear closely linked to underlying patient characteristics, HF phenotypes, and the organizational pathways through which care is delivered. Building on these findings, patients in this study were characterized by advanced age, high comorbidity burden, and a predominance of HFpEF, features known to influence therapeutic decisions and outcomes [16,17]. Such complexity often necessitates individualized management strategies that go beyond conventional guideline frameworks.

Frailty, multimorbidity, renal dysfunction, and polypharmacy may shape treatment intensity and tolerance, contributing to the variability observed in GDMT use [25,26,27,28,29,30,31]. In patients with HFpEF, highly prevalent in our cohort, evidence supporting specific therapies remains limited, which may further explain treatment differences [32].

### 4.1. Interpretation of Admission Pathways

The admission service typically reflects each patient’s initial clinical profile and care needs. IM tends to manage older, frailer individuals with multiple comorbidities, while CAR often admits patients with more overt cardiac disease or requiring specialized diagnostics [16,20,25]. These structural and organizational factors help contextualize the observed therapeutic patterns and outcomes. Understanding these differences provides important context for interpreting comparative data across settings.

Patients admitted to IM were older, predominantly female, and more likely to have HFpEF. By contrast, those admitted to CAR were younger, more often male, and received markedly higher rates of GDMT, including RAAS inhibitors, β-blockers, MRAs, and SGLT2 inhibitors.

The higher adjusted mortality observed among patients admitted under IM in our dataset is in accordance with the prior literature showing that older, more comorbid patients experience worse survival, regardless of care setting [20,21,22,23]. Prior European studies have shown that patients cared for by internists are older, frailer, and more comorbid, with substantially lower rates of evidence-based therapies compared with those managed in cardiology wards [4,8,12,25,26,27]. Similar patterns have been reported in other real-world international registries, including the ESC-HF Long-Term Registry and the EuroHeart Failure Surveys, which revealed wide variability in HF management across regions and hospital settings [7,10,26]. These studies highlight a recurring gap between guideline recommendations and real-world practice, particularly in frail or multimorbid populations, such as the elderly HF patients. This finding underscores the importance of integrating geriatric assessment, optimizing GDMT when appropriate, and coordinating follow-up care across services. The absence, in our sample, of a comprehensive assessment of comorbidities and a standardized frailty score highlights the importance of systematically incorporating these variables to enable a more complete characterization of the study population and to derive conclusions that more accurately reflect real-world conditions.

Although readmission rates were similar in our study, the survival disadvantage among IM patients suggests that the gap is primarily driven by baseline vulnerability and differences in therapeutic implementation rather than by readmission burden alone. Importantly, our study extends previous evidence by focusing exclusively on the very elderly, a population largely excluded from major clinical trials and underrepresented in registries [10,11,12,13].

Fragility, comorbidities, and social context must be considered when managing patients and used to adapt interventions to the patient’s needs, reducing the gap between clinical practice and guideline recommendations in the elderly population.

### 4.2. Potential Mechanisms and Explanations

The higher mortality observed among IM patients should be interpreted cautiously and is likely multifactorial rather than attributable to a single mechanism. In our cohort, IM patients were significantly older and had a greater burden of comorbidities, including renal dysfunction, factors that have been associated with poorer survival, functional decline, and reduced tolerance of HF therapies [16,28]. In older patients, polypharmacy, frailty, renal impairment, and concerns about adverse events may influence clinicians’ decisions regarding initiation or up-titration of disease-modifying therapies; however, this interpretation remains hypothesis-generating, as our data do not allow us to determine whether lower treatment rates reflected therapeutic inertia, clinical contraindications, patient frailty, or limited tolerability [29,30]. Importantly, contemporary evidence suggests that advanced age and renal dysfunction should not, by themselves, preclude the use of HF pharmacotherapy when clinically indicated and appropriately monitored. A recent systematic review and network meta-analysis of modern HFrEF therapies in specific patient subgroups showed that sacubitril/valsartan, dapagliflozin, and empagliflozin were associated with reductions in the composite endpoint of cardiovascular death or HF hospitalization in older patients, while sacubitril/valsartan, dapagliflozin, and vericiguat showed benefit in patients with chronic kidney disease [31]. These data support efforts to optimize guideline-directed medical therapy in high-risk subgroups, while individualizing treatment according to renal function, frailty, comorbidity burden, and patient tolerance. Nevertheless, because IM patients in our study were more frequently diagnosed with HFpEF, the lower use of some GDMT components may also reflect differences in the strength of evidence and recommendations across HF phenotypes rather than undertreatment alone [20,21,22,32,33,34]. Recent evidence has strengthened the role of SGLT2 inhibitors beyond HFrEF, particularly in HFmrEF/HFpEF, although the evidence base for other disease-modifying therapies remains less robust than in HFrEF. Therefore, the association between IM status and mortality should be regarded as clinically relevant but not necessarily mechanistically causal.

### 4.3. Sex Differences

Sex differences emerged as another key dimension in our findings. Women who represented nearly two-thirds of our cohort were older and more often diagnosed with HFpEF. These trends echo prior studies showing that women with HF are less likely to receive RAAS inhibitors, β-blockers, MRAs, and SGLT2 inhibitors, even when clinically eligible [17,18,26,35]. Although sex-specific higher prevalence of HF remains inconclusive [36], these differences have therapeutic implications, since women with HFpEF remain underrepresented in clinical trials [18,22,37,38].

There were no significant differences between sexes in all-cause mortality, HF readmission and in both endpoints combined, although a non-significant trend towards a higher rate of readmissions in women was observed. These sex differences may reflect biological and social determinants of health: women with HFpEF tend to live longer but experience more symptomatic relapses and functional decline, whereas men with HFrEF face higher early mortality [39]. These findings reinforce the need for sex-specific management strategies that address the unique vulnerabilities of each group.

### 4.4. Clinical and Health System Implications

The implications of our study results extend beyond individual care to the structure of health systems. Standardized, multidisciplinary pathways are needed to harmonize care across departments. The establishment of integrated HF units, combining the expertise of cardiology, internal medicine, geriatrics, and nursing teams, has been shown to enhance adherence to guidelines and reduce hospitalizations [21,40]. Moreover, telemonitoring and nurse-led titration programs represent promising tools for extending specialist support beyond hospital walls, particularly for older patients with limited mobility or access [41]. Addressing care fragmentation is crucial in this context to achieve a cohesive approach to managing HF patients [34].

Given the predominance of HFpEF in our cohort, management strategies must also address comorbidities, such as hypertension, diabetes, anemia, and chronic kidney disease, and incorporate functional and social determinants of health. Wider adoption of the latest therapies, such as SGLT2 inhibitors across the entire EF spectrum, could meaningfully improve outcomes in this high-risk group [21].

### 4.5. Strengths, Limitations, and Future Directions

This study focuses on an underrepresented age group and a real-world assessment of treatment and outcomes across clinical services. Nonetheless, limitations must be acknowledged. The retrospective observational design precludes causal inference, and residual confounding cannot be excluded. We did not capture adherence after discharge or the quality of follow-up care, both of which may have influenced outcomes. Data on frailty indices were not available. The study did not consider comorbidity indices, so we cannot rule out residual confounding. Furthermore, results may not be generalizable to countries with different healthcare structures. Due to the limited number of patients with all data available, the conclusions of multivariate analyses are limited. Despite these caveats, the consistency of our findings across multiple analyses reinforces their validity and clinical relevance.

Future research should prioritize pragmatic, implementation-focused trials in the populations most affected by care disparities, namely, elderly women with HFpEF and renal dysfunction. Identifying the barriers to GDMT prescription in real-world settings and testing interventions that improve uptake will be critical. Integration of geriatric assessment into HF management, alongside interdisciplinary collaboration between cardiology, internal medicine, and primary care, represents an essential step toward personalized, equitable, and sustainable care for this expanding population.

Another limitation of our study is the absence of a formal frailty assessment. Although frailty is particularly relevant in this patient population, conventional clinical frailty scales were not available. In addition, biochemical frailty scores, such as the biological FRAil score [42], could not be reliably calculated because the required biochemical variables were not systematically collected. Future studies should prospectively incorporate clinical or biochemical frailty assessments to better define the clinical profile of these patients and evaluate their potential additive prognostic value.

## 5. Conclusions

Among adults aged ≥80 years hospitalized for acute HF, we observed distinct clinical profiles, treatment patterns, and outcomes that varied according to patient characteristics, HF phenotype, and hospital admission pathways. These findings underscore the importance of coordinated, sex-sensitive, and multidisciplinary HF care models tailored to the complex needs of an aging population.

## Figures and Tables

**Table 1:** Baseline characteristics of hospitalized patients, stratified by admission service and sex.

	Admission Service				Sex		
Variable	Overall (*n* = 1226)	CAR (*n* = 359)	IM (*n* = 867)	*p*-Value	Women (*n* = 807)	Men (*n* = 419)	*p*-Value
Age, years	87 [7]	85 [6]	88 [7]	<0.001 ***	88 [6]	86 [7]	<0.001 ***
Sex (male)	419 (34.2)	150 (41.8)	269 (31.0)	<0.001 ***	-	-	-
SBP (mmHg)	136 [35]	136 [34]	136 [35]	0.21	137 [35]	133 [33]	0.006 **
DBP (mmHg)	74 [26]	75 [27]	73 [25]	0.22	74 [28]	73 [23]	0.28
HFrEF	139 (20.0)	88 (27.2)	51 (13.7)	<0.001 ***	51 (11.8)	88 (33.6)	0.001 ***
HFmrEF	91 (13.1)	46 (14.2)	45 (12.1)		48 (11.1)	43 (16.4)	
HFpEF	466 (67)	189 (58.5)	277 (74.3)		335 (77.2)	131 (50)	
Diabetes mellitus	529 (43.2)	148 (41.5)	381 (43.9)	0.42	318 (39.4)	211 (50.6)	<0.001 ***
Hypertension	1050 (85.8)	304 (85.2)	746 (86.0)	0.68	692 (85.7)	358 (85.9)	0.96
Ischemic cardiopathy	255 (21.4)	93 (26.9)	162 (19.1)	0.003 **	138 (17.5)	117 (29.0)	<0.001 ***
History of AF	629 (51.3)	173 (48.9)	456 (52.8)	0.20	433 (53.9)	196 (47.3)	0.03 *
Previous admissions due to HF	555 (45.6)	147 (41.4)	408 (47.3)	0.06	392 (48.8)	163 (39.4)	0.002 **
NYHA > II	613 (52.1)	133 (39.6)	480 (57.1)	<0.001 ***	425 (54.6)	188 (47.1)	0.015 *
RF (mL/min/1.73 m^2^)	46 [33]	49 [30]	45 [34]	0.13	45 [33]	48 [33]	0.034 *
HF etiology							
Ischemic	121 (10.0)	55 (15.3)	66 (7.7)	<0.001 ***	58 (7.3)	63 (15.1)	<0.001 ***
Valvular	312 (25.7)	128 (35.7)	184 (21.5)		222 (27.9)	90 (21.6)	
Hypertensive	134 (11.0)	41 (11.4)	93 (10.9)		96 (12.0)	38 (9.1)	
Unknown	234 (19.3)	30 (8.4)	204 (23.9)		172 (21.6)	148 (35.6)	
Other	392 (32.3)	96 (26.7)	296 (34.7)		244 (30.6)	148 (35.6)	

Values are expressed as median [interquartile range] (*p*-value by Mann–Whitney) and (%) (*p*-value by chi-square test). *p*-value: * *p* < 0.05; ** *p* < 0.01; *** *p* < 0.001. Abbreviations: AF: atrial fibrillation; CAR: cardiology service; DBP: diastolic blood pressure; HF: heart failure; HFrEF: HF with reduced ejection fraction; HFmrEF: HF with mildly reduced ejection fraction; HFpEF: HF with preserved ejection fraction; IM: internal medicine service; NYHA: New York Heart Association; RF: renal function measured as Modification of Diet in Renal Disease equation-4; SBP: systolic blood pressure.

**Table 2:** Discharge therapies in patients, stratified by admission service and sex.

		Admission Service			Sex		
Drug Class	Overall (*n* = 1226)	CAR (*n* = 359)	IM (*n* = 867)	*p*-Value	Women (*n* = 807)	Men (*n* = 419)	*p*-Value
β-blockers	653 (53.3)	255 (71.0)	398 (45.9)	<0.001 ***	451 (55.9)	202 (48.2)	0.01 **
ACEi	153 (12.5)	48 (13.4)	105 (12.1)	0.54	104 (12.9)	49 (11.7)	0.54
ARBs	282 (23.0)	86 (24.0)	196 (22.6)	0.61	209 (25.9)	73 (17.4)	<0.001 ***
ARNis	66 (5.4)	46 (12.8)	20 (2.3)	<0.001 ***	28 (3.5)	38 (9.1)	<0.001 ***
MRAs	240 (19.6)	104 (29.0)	136 (15.7)	<0.001 ***	155 (19.2)	85 (20.3)	0.65
SGLT2is	169 (13.8)	91 (25.3)	78 (9.0)	<0.001 ***	101 (12.5)	68 (16.2)	0.74
Loop diuretics	887 (72.3)	316 (88.0)	571 (65.9)	<0.001 ***	593 (73.5)	294 (70.2)	0.21
Statins	488 (39.8)	208 (57.9)	280 (32.3)	<0.001 ***	300 (37.2)	188 (44.9)	0.009 **
Antiplatelet drugs	226 (18.4)	99 (27.6)	127 (14.6)	<0.001 ***	129 (16.0)	97 (23.2)	0.002 **
Anticoagulants	546 (44.5)	199 (55.4)	347 (40.0)	<0.001 ***	369 (45.7)	177 (42.2)	0.24
Calcium antagonists	221 (18,0)	68 (18.9)	153 (17.6)	0.59	147 (18.2)	74 (17.7)	0.81

Values are expressed as *n* (number of patients) and % with respect to each sex or admission service group. *p*-values by chi-square test. *p*-value: ** *p* < 0.01; *** *p* < 0.001. Abbreviations: ACEi: angiotensin-converting enzyme inhibitor; ARBs: angiotensin II receptor blockers; ARNis: angiotensin receptor–neprilysin inhibitors; CAR: cardiology service; IM: internal medicine service; MRAs: mineralocorticoid receptor antagonists; SGLT2is: sodium–glucose cotransporter-2 inhibitors.

**Table 3:** Discharge medication stratified by sex in within each admission service.

Drug Class	Women in CAR (*n* = 209)	Men in CAR (*n* = 150)	*p*-Value ^b^	Women in IM (*n* = 598)	Men in IM (*n* = 269)	*p*-Value ^a^	*p*-Value (All Groups)
β-blockers	159 (76.1)	96 (64.0)	0.01	292 (48.8)	106 (39.4)	0.01 **	<0.001 ***
ACEi	30 (14.4)	18 (12.0)	0.51	74 (12.4)	31(11.5)	0.72	0.81
ARBs	58 (27.8)	28 (18.7)	0.04	151 (25.3)	45 (16.7)	0.006 **	0.008 **
ARNis	18 (8.6)	28 (18.7)	0.005	10 (1.7)	10 (3.7)	0.06	<0.001 ***
MRAs	51 (24.4)	53 (35.3)	0.002	104 (17.4)	32 (11.9)	0.04 *	<0.001 ***
SGLT2is	49 (23.4)	42 (28.0)	0.32	52 (8.7)	26 (9.7)	0.64	<0.001 ***
Loop diuretics	185 (88.5)	131 (87.3)	0.73	408 (68.2)	163 (60.6)	0.03 *	<0.001 ***
Statins	107 (51.2)	101 (67.3)	0.02	193 (32.3)	87 (32.3)	0.98	<0.001 ***
Antiplatelet drugs	49 (23.4)	50 (33.3)	0.04	80 (13.4)	47 (17.5)	0.11	<0.001 ***
Anticoagulants	124 (59.3)	75 (50.0)	0.08	245 (41.0)	102 (37.9)	0.40	<0.001 ***
Calcium antagonists	44 (21.1)	24 (16.0)	0.23	103 (17.2)	50 (18.6)	0.40	0.56

Values are expressed as *n* (number of patients) and % with respect to each sex group. *p*-values by chi-square test. *p*-value: * *p* < 0.05; ** *p* < 0.01; *** *p* < 0.001. Abbreviations: ACEi: angiotensin-converting enzyme inhibitor; ARBs: angiotensin II receptor blockers; ARNis: angiotensin receptor–neprilysin inhibitors; CAR: cardiology service; IM: internal medicine service; MRAs: mineralocorticoid receptor antagonists; SGLT2is: sodium–glucose cotransporter-2 inhibitors. ^a^ women vs. men in IM. ^b^ women vs. men in CAR.

**Table 4:** Discharge therapies, stratified by admission service and sex, in patients with HFrEF (*n* = 139 patients).

		Admission Service			Sex		
Drug Class	Overall (*n* = 139)	CAR (*n* = 88)	IM (*n* = 51)	*p*-Value	Women (*n* = 51)	Men (*n* = 88)	*p*-Value
β-blockers	92 (66.2)	72 (70.5)	30 (58.8)	0.16	32 (62.7)	60 (68.28)	0.51
ACEi	15 (10.8)	11 (12.58)	4 (7.8)	0.39	7 (13.8)	8 (9.1)	0.39
ARBs	15 (10.8)	8 (9.1)	7 (13.7)	0.39	9 (17.6)	6 (6.8)	0.04 *
ARNis	42 (30.2)	34 (38.6)	8 (15.7)	0.005 **	8 (15.7)	34 (38.6)	0.005 **
MRAs	56 (40.3)	47 (53.4)	9 (17.6)	<0.001 ***	17 (33.3)	39 (44.3)	0.20
SGLT2is	33 (23.7)	25 (28.47)	8 (15.7)	0.09	8 (15.7)	25 (28.4)	0.09
Loop diuretics	109 (78.4)	74 (84.1)	35 (68.6)	0.003 *	38 (74.5)	71 (80.7)	0.39
Statins	73 (52.5)	54 (61.4)	19 (37.3)	0.006 **	18 (35.3)	55 (62.5)	0.002 **
Antiplatelet drugs	47 (33.8)	32 (36.4)	15 (29.4)	0.40	8 (15.7)	39 (44.3)	<0.001 ***
Anticoagulants	68 (48.9)	46 (52.3)	22 (43.1)	0.29	29 (56.9)	39 (44.3)	0.15
Calcium antagonists	13 (9.4)	3 (3.4)	10 (3.4)	0.002 **	2 (3.9)	11 (12.5)	0.09

Values are expressed as *n* (number of patients) and % with respect to each sex or admission service group. *p*-values by chi-square test. *p*-value: * *p* < 0.05; ** *p* < 0.01; *** *p* < 0.001. Abbreviations: ACEi: angiotensin-converting enzyme inhibitor; ARBs: angiotensin II receptor blockers; ARNis: angiotensin receptor–neprilysin inhibitors; CAR: cardiology service; HFrEF: heart failure with reduced ejection fraction; IM: internal medicine service; MRAs: mineralocorticoid receptor antagonists; SGLT2is: sodium–glucose cotransporter-2 inhibitors.

**Table 5:** Discharge therapies, stratified by admission service and sex, in patients with HFmrEF (*n* = 91 patients).

		Admission Service			Sex		
Drug Class	Overall (*n* = 91)	CAR (*n* = 46)	IM (*n* = 45)	*p*-Value	Women (*n* = 48)	Men (*n* = 43)	*p*-Value
β-blockers	59 (64.8)	38 (82.6)	21 (46.7)	<0.001 ***	38 (79.2)	21 (48.8)	0.002 **
ACEi	16 (17.1)	11 (23.9)	5 (11.1)	0.11	9 (18.8)	7 (16.3)	0.75
ARBs	30 (33)	18 (39.1)	12 (26.7)	0.21	19 (39.6)	11 (25.6)	0.15
ARNis	6 (6.6)	4 (8.7)	2 (4.4)	0.41	5 (10.4)	1 (2.3)	0.12
MRAs	28 (30.8)	19 (41.3)	9 (20)	0.03 *	16 (33.3)	12 (27.9)	0.57
SGLT2is	15 (16.5)	13 (28.3)	2 (4.4)	0.002 **	8 (16.7)	7 (16.3)	0.96
Loop diuretics	70 (76.9)	41 (89.1)	29 (64.4)	0.005 **	40 (83.3)	30 (69.8)	0.12
Statins	50 (54.9)	31 (67.4)	19 (42.2)	0.01 **	27 (56.3)	23 (53.5)	0.79
Antiplatelet drugs	20 (22)	13 (28.3)	7 (15.6)	0.14	10 (20.8)	10 (23.3)	0.78
Anticoagulants	46 (50.5)	25 (54.3)	21 (46.7)	0.46	29 (60.4)	17 (39.5)	0.04 *
Calcium antagonists	16 (17.6)	11 (23.9)	5 (11.1)	0.11	7 (14.6)	9 (20.9)	0.43

Values are expressed as *n* (number of patients) and % with respect to each sex or admission service group. *p*-values by chi-square test. *p*-value: * *p* < 0.05; ** *p* < 0.01; *** *p* < 0.001. Abbreviations: ACEi: angiotensin-converting enzyme inhibitor; ARBs: angiotensin II receptor blockers; ARNis: angiotensin receptor–neprilysin inhibitors; CAR: cardiology service; HFmrEF: heart failure with mildly reduced ejection fraction; IM: internal medicine service; MRAs: mineralocorticoid receptor antagonists; SGLT2is: sodium–glucose cotransporter-2 inhibitors.

**Table 6:** Discharge therapies, stratified by admission service and sex, in patients with HFpEF (*n* = 466 patients).

		Admission Service			Sex		
Drug Class	Overall (*n* = 466)	CAR (*n* = 189)	IM (*n* = 277)	*p*-Value	Women (*n* = 335)	Men (*n* = 131)	*p*-Value
β-blockers	291 (62.4)	137 (72.5)	154 (55.6)	<0.001 ***	220 (65.7)	71 (54.2)	0.02 *
ACEi	62 (13.3)	22 (11.6)	40 (14.4)	0.38	45 (13.4)	17 (13)	0.89
ARBs	128 (27.5)	53 (28)	75 (27.1)	0.82	99 (29.6)	29 (22.1)	0.11
ARNis	6 (1.3)	4 (2.1)	2 (0.7)	0.19	5 (1.5)	1 (0.8)	0.53
MRAs	74 (15.9)	28 (14.8)	46 (16.6)	0.60	57 (17)	17 (13)	0.28
SGLT2is	79 (17)	43 (22.8)	36 (13)	0.006 **	58 (17.3)	21 (16)	0.74
Loop diuretics	379 (81.3)	174 (92.1)	205 (74)	<0.001 ***	272 (81.2)	107 (81.7)	0.90
Statins	210 (45.1)	108 (57.1)	102 (36.8)	<0.001 ***	145 (43.3)	65 (49.6)	0.22
Antiplatelet drugs	84 (18)	47 (24.9)	37 (13.4)	0.002 **	59 (17.6)	25 (19.1)	0.71
Anticoagulants	240 (51.5)	111 (58.7)	129 (46.6)	0.01 **	174 (51.9)	66 (50.4)	0.76
Calcium antagonists	112 (24)	46 (24.3)	66 (23.8)	0.90	84 (25.1)	28 (21.4)	0.40

Values are expressed as *n* (number of patients) and % with respect to each sex or admission service group. *p*-values by chi-square test. *p*-value: * *p* < 0.05; ** *p* < 0.01; *** *p* < 0.001. Abbreviations: ACEi: angiotensin-converting enzyme inhibitor; ARBs: angiotensin II receptor blockers; ARNis: angiotensin receptor–neprilysin inhibitors; CAR: cardiology service; HFpEF: heart failure with preserved ejection fraction; IM: internal medicine service; MRAs: mineralocorticoid receptor antagonists; SGLT2is: sodium–glucose cotransporter-2 inhibitors.

**Table 7:** Predictive model of the presence of outcomes during follow-up by sex and admission service.

12-Month Follow-Up Outcomes			
	**HR**	**95% CI**	***p*** **-Value**
EFFECT OF SEX ON OUTCOMES			
All-cause death	0.91	0.68–1.22	0.56
First HF readmission *	0.51	0.25–1.05	0.07
Composite event (all-cause death or first-time readmission)	1.03	0.78–1.36	0.80
EFFECT OF ADMISSION SERVICE ON OUTCOMES			
All-cause death	1.91	1.42–2.60	<0.001
First HF readmission *	1.63	0.78–3.42	0.20
Composite event (all-cause death or first-time readmission)	1.82	1.38–2.42	<0.001

Cox regression model adjusted by age, renal function, left ventricular ejection fraction, NYHA (I or II vs. III or IV), ischemic etiology, diabetes mellitus, systolic blood pressure, previous history of atrial fibrillation and previous HF admission. * Adjusted by the Fine–Gray method. CI: confidence interval; HF: heart failure; HR: hazard ratio.

## Data Availability

The original contributions presented in this study are included in the article. Further inquiries can be directed to the corresponding authors.

## Abbreviations

The following abbreviations are used in this manuscript:

ARNis	angiotensin receptor–neprilysin inhibitors
CAR	cardiology
CI	confidence interval
GDMT	guideline-directed medical therapy
HF	heart failure
HFpEF	heart failure with preserved ejection fraction
HFmrEF	heart failure with mildly reduced ejection fraction
HFrEF	heart failure with reduced ejection fraction
HR	hazard ratio
IM	internal medicine
LVEF	left ventricular ejection fraction
MRAs	mineralocorticoid receptor agonists
NYHA	New York Heart Association
RAAS	renin–angiotensin–aldosterone system
SGLT2	sodium–glucose cotransporter-2
